# Brain-enriched miR-128: Reduced in exosomes from Parkinson’s patient plasma, improves synaptic integrity, and prevents 6-OHDA mediated neuronal apoptosis

**DOI:** 10.3389/fncel.2022.1037903

**Published:** 2023-01-12

**Authors:** Pallabi Bhattacharyya, Atanu Biswas, Subhas C. Biswas

**Affiliations:** ^1^Cell Biology and Physiology Division, CSIR-Indian Institute of Chemical Biology, Kolkata, India; ^2^Department of Neurology, Bangur Institute of Neurosciences, Kolkata, India

**Keywords:** miR-128, Parkinson’s disease, brain-enriched miRNAs, neuron-enriched miRNAs, apoptosis, Fas–ligand, PUMA (p53 upregulated modulator of apoptosis), FoxO3a

## Abstract

Parkinson’s disease (PD) is a progressive neurodegenerative disorder associated with the death of mid-brain dopaminergic neurons. Unfortunately, no effective cure or diagnostic biomarkers for PD are available yet. To address this, the present study focuses on brain-enriched small non-coding regulatory RNAs called microRNAs (miRNAs) that are released into the circulation packaged inside small extracellular vesicles called exosomes. We collected blood samples from PD patients and isolated exosomes from the plasma. qPCR-based detection revealed a particular neuron-enriched miR-128 to be significantly decreased in the patient-derived exosomes. Interestingly, a concomitant decreased expression of miR-128 was observed in the cellular models of PD. Fluorescent live cell imaging and flow-cytometry revealed that over-expression of miR-128 can prevent 6-OHDA-mediated mitochondrial superoxide production and induction of neuronal death respectively. This neuroprotective effect was found to be induced by miR-128-mediated inhibition of FoxO3a activation, a transcription factor involved in apoptosis. miR-128 over-expression also resulted in down-regulation of pro-apoptotic FoxO3a targets- FasL and PUMA, at both transcript and protein levels. Further downstream, miR-128 over-expression inhibited activation of caspases-8, -9 and -3, preventing both the intrinsic and extrinsic pathways of apoptosis. Additionally, over expression of miR-128 prevented down-regulation of synaptic proteins- Synaptophysin and PSD-95 and attenuated neurite shortening, thereby maintaining overall neuronal integrity. Thus, our study depicts the intracellular role of miR-128 in neuronal apoptosis and neurodegeneration and its implications as a biomarker being detectable in the circulating exosomes of PD patient blood. Thus, characterization of such exosomal brain-enriched miRNAs hold promise for effective detection and diagnosis of PD.

## Graphical Abstract

Schematic representation of the role of brain-enriched miR-128 in Parkinson’s disease pathogenesis and progression. 
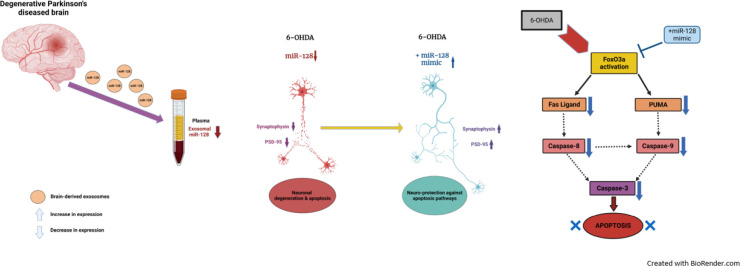

## Introduction

Parkinson’s disease (PD), a progressive age-related neurodegenerative disorder, is mediated by the loss of dopaminergic neurons in the mid-brain (Bekris et al., 2010; Lin and Farrer, 2014). It is primarily characterized by motor dysfunctions namely muscle rigidity, bradykinesia, postural instability and tremor although non-motor manifestations like sleep disorder, fatigue, depression, gastrointestinal or sexual dysfunctions are also not uncommon (Bloem et al., 2021). Incidentally, more than 200 years have passed since the identification of PD by James Parkinson, in 1817 (Obeso et al., 2017), but unfortunately, any cure or biomarker for its early detection are not available till date. Our present study aims at targeting this scientific lacuna by focusing on a category of small non-coding RNAs called microRNAs (miRNAs) that may be released into the circulatory system from the brain tissue, during pathogenesis of PD.

miRNAs (20–23 nucleotides) are important regulators of gene expression, typically at the posttranscriptional level (Filipowicz et al., 2008; Bhattacharyya and Biswas, 2020). Interestingly, miRNA expression, itself, is temporally and spatially controlled, with some miRNAs known to be expressed ubiquitously, while certain others are expressed preferentially in specific cell or tissue types. Incidentally, there are specific miRNAs which are brain-enriched and are preferentially expressed in particular brain cells like neurons or glia, suggesting their important regulatory functions in the pathogenesis of brain-related disorders like PD. Additionally, miRNAs may be detected extracellularly, in circulating body fluids such as blood, saliva, urine and CSF, making them important targets as biomarkers for brain diseases, including PD. There has been a plethora of work identifying a wide range of differentially expressed circulating miRNAs associated with PD, but none of them have been successful as a biomarker so far. One of the biggest caveats of such studies, we believe, is the source of miRNAs under study. While the circulating miRNAs may show a dysregulated pattern, these ubiquitously expressed miRNAs could be derived from any tissue or cell type and may not be specific to the particular tissue under consideration (in this case, brain). This makes reproducibility a major problem, as the miRNA profile may vary from subject to subject. Secondly, the type of cell-free miRNAs to be identified also needs careful consideration. It is worth noting here that miRNAs may be released by the parental cells or tissue in two major ways either as a ribonucleoprotein complex associated with Argonaute proteins, or encapsulated within membrane-bound extracellular vesicles (EVs) like apoptotic bodies, shedding vesicles and exosomes (Turchinovich et al., 2012). Among these, exosomes are the most commonly identified small extracellular vesicles (sEV) to carry miRNAs in the circulating body fluids.

Exosomes (40–160 nm diameter), are most commonly released by the cells as means of secreting cargo (miRNAs, in this case) (Kalluri and LeBleu, 2020). These exosomes may be uptaken by the neighboring cells, thereby facilitating cell-to-cell communication (Valadi et al., 2007; Desrochers et al., 2016). Additionally, exosomes are released by the parental cells or tissue in an energy-dependent manner, through specific signaling cascades that maybe dysregulated during disease pathogenesis (Turchinovich et al., 2012). Thus, the exosomal miRNA profile would better reflect the altered intracellular states under diseased conditions, in contrast to the cell-free miRNAs that maybe released as a passive by-product of cell lysis, not particularly indicative of the diseased state (Gurung et al., 2021). Further, exosomes are specifically important in context to brain diseases as they are known to cross the blood-brain-barrier effectively (Haney et al., 2015; Banks et al., 2020), thereby allowing the brain-derived miRNAs to come into the circulation.

Taking into consideration the above factors, we have hypothesized that identification and characterization of specific brain-enriched exosome-derived miRNA signatures are essential for understanding PD pathogenesis and could be more appropriate candidates as PD biomarkers. Through extensive literature review, we narrowed down to a particular brain- and neuron-enriched miRNA, miR-128 (Smirnova et al., 2005; Shao et al., 2010; Liu and Xu, 2011; He et al., 2012), which seemed to be an interesting candidate for our study on PD. miR-128 first came into prominence when Tan et al. (2013) identified that miR-128 deficiency can cause motor dysfunction and seizure- induced death in epileptic mouse models. On the other hand, ectopic expression of miR-128 was found to restore excitability in post-natal dopamine responsive (D1) neurons. Since then, the significance of miR-128 in different motor neuron function and diseases as a “motomiR” has been explored (Hawley et al., 2017). Interestingly, a recent report has detected decreased levels of miR-128 in the CSF of PD patients (Burgos et al., 2014; van den Berg et al., 2020) although the mechanistic implications of miR-128 in PD pathogenesis, if any, is still largely unexplored.

In the present study, we identified the expression of miR-128 to be characteristically reduced in the circulating exosomes derived from the blood (plasma) samples of a PD patient cohort. Since miR-128 is a neuron-enriched miRNA, we decided to corroborate whether the altered extracellular expression pattern of the miRNA is also associated with dysregulated intracellular diseased states in cellular models of PD. We found that miR-128 can prevent 6-OHDA-mediated neuronal death by inhibiting both the intrinsic and extrinsic pathways of apoptosis downstream of the activation of the transcription factor, FoxO3a. miR-128 was also instrumental in maintaining neurite formation and synaptic integrity, showing neuroprotective functions overall.

## Materials and methods

### Human blood sample collection and plasma separation

Blood samples were collected at the Bangur Institute of Neurosciences (BIN), Kolkata, India, from PD patients (*n* = 25) and respective age-matched controls (*n* = 20), in EDTA-vials (BD Biosciences). The details of the human subjects are tabulated in Supplementary Table 4. The upper plasma layer was separated from the blood samples by centrifugation at 2,000×*g* for 10 min at 4^°^C.

### Exosome isolation

Exosomes were separated from the human plasma samples by miRCURY Exosome Serum/Plasma kit (Qiagen) as per manufacturer’s protocol. Briefly, plasma samples were incubated with Thrombin at room temperature for 5 min and then centrifuged at 10,000×*g* for 5 min. The supernatant was collected and Precipitation Buffer was added to it. The sample was allowed to incubate overnight at 4^°^C. It was then centrifuged at 500×g for 5 min at 20^°^C. Resuspension buffer was added to the pellet and this purified exosome sample was used for further downstream processing.

### Nano-particle tracking analysis of exosomes

Purified exosomes were 10-fold diluted in 1X PBS and subjected to nano-particle tracker analysis (NTA) (NanoSight NS300) as per manufacturer’s guidelines.

### Cell culture and 6-OHDA treatment

The human neuroblastoma cell line SH-SY5Y (NCCS, Pune) was maintained in DMEM medium (Gibco) supplemented with 10% heat-inactivated fetal bovine serum/FBS (Gibco) and differentiated for 5–7 days in the same medium supplemented with all-trans retinoic acid/ATRA (10 μM) (Sigma). The rat pheochromocytoma cell line PC12 was maintained in DMEM medium supplemented with 10% heat-inactivated horse serum/HS (Gibco) and 5% FBS. It was differentiated in DMEM medium supplemented with 1% HS and nerve growth factor/NGF - β (50 ng/mL) (Sigma) for 5–7 days.

6-OHDA or 6-hydroxydopamine (Sigma) was dissolved in DMEM medium and added to the cells *in vitro* in indicated concentrations for respective time-points as mentioned in the experiments.

### RNA isolation and quantitative real-time PCR

RNA isolation was done using TRIzol Reagent (Thermo Fisher) as per phenol-chloroform based total RNA extraction method. For miRNA analysis, cDNA was prepared using TaqMan MicroRNA Reverse Transcription Kit (Thermo Fisher) using manufacturer’s protocol, followed by qRT-PCR using TaqMan Universal PCR Master Mix (Thermo Fisher) and specific miRNA assay reagents (Thermo Fisher) as enlisted in Supplementary Table 1. For mRNA analysis, cDNA was prepared using PrimeScript 1st strand cDNA Synthesis Kit (Takara Bio) using manufacturer’s protocol, followed by qRT-PCR using SYBR Green PCR Master Mix (Thermo Fisher). The primers used for mRNA qRT-PCR based analysis are enlisted in Supplementary Table 2. U6 snRNA and GAPDH mRNA were used as endogenous controls in miRNA and mRNA qRT-PCR analysis respectively. All qRT-PCR reactions were done in StepOnePlus Real-Time PCR System (Thermo Fisher). Comparative C_T_ Method (ΔΔC_T_ Method) was used for the qRT-PCR data analysis.

### miRNA mimic transfection

SH-SY5Y or PC12 cells were plated and primed for 2 days in the presence of ATRA or NGF-β respectively. Transfection with miRNA mimic and negative control mimic (Ambion) (Supplementary Table 1) was done on the second day of priming in Opti-MEM medium (Thermo Fisher) using Lipofectamine RNAiMAX Reagent (Invitrogen), following manufacturer’s protocol. Six hours post-transfection, Opti-MEM medium was replaced with the respective priming medium. Cells were further maintained for 48 h and then treated with 6-OHDA on the fifth day of priming.

### Flow cytometry

Cells were analyzed by flow cytometry using LIVE/DEAD Viability/Cytotoxicity Kit for mammalian cells (Thermo Fisher), using manufacturer’s protocol. Briefly, cells *in vitro* were trypsinized and a cell suspension was prepared in culture medium. Calcein-AM and ethidium homodimer-1 was added to the cell suspension and incubated at room temperature for 15–20 min in dark. The stained cells were analyzed by flow cytometry using 488 nm excitation, detecting green fluorescence emission for Calcein AM (i.e., 530/30 bandpass) and red fluorescence emission for Ethidium homodimer-1 (i.e., 610/20 bandpass). The cells were gated on to exclude debris. Standard compensation was done using single color stained cells. The population of cells were separated into two groups: live cells with green fluorescence and dead cells with red fluorescence. BD LSRFortessa was used for all flow cytometry experiments.

### Live-cell imaging for mitochondrial superoxide detection

Cells *in vitro* were incubated with MitoSOX Red reagent (Thermo Fisher) prepared in HBSS buffer (Ca^2+^, Mg^2+^, no phenol red) for 10 min in dark in the humidified cell culture incubator (37^°^C, 5% CO2). Cells were washed with warm buffer and imaged under fluorescent microscope (Leica) under 20X objectives with excitation at 510 nm and emission at 580 nm.

### Immunoblotting

Cultured cells *in vitro* were lysed in RIPA buffer (Thermo Scientific) supplemented with ProteoGuard EDTA-Free Complete Protease Inhibitor Cocktail (Takara) and protein samples (25–50 μg) from whole cell lysates were resolved by SDS-PAGE. Gels were transferred on PVDF membranes (GE Healthcare) at 100 V for 1–2 h at 4^°^C. Membranes were blocked in 5% non-fat dry milk or 5% bovine serum albumin (BSA) for 1 h at room temperature. Primary antibodies were diluted in the blocking solution (Supplementary Table 3) and incubated overnight at 4^°^C. HRP-conjugated secondary antibodies diluted in blocking solution were used against the respective primary antibodies and incubated at room temperature for 1 h. Detection of protein bands was carried out by using Clarity Max Western ECL substrate (Bio-Rad) as per manufacturer’s protocol. Images of all the blots were taken using iBright Imaging system (Thermo Fisher).

### Neurite length measurement

Primed PC12 cells were observed under bright field microscope using 20X objectives. Neurite length was measured using the NeuronJ plugin of Fiji software (Popko et al., 2009).

### Schematic representation

All the schematic diagrams were prepared using BioRender.com.

### Statistical analysis

All graphs, statistical analyses and image analyses were done using GraphPad Prism 9.0, ImageJ and Fiji softwares. One-way ANOVA with Tukey correction was used to analyze data concerning comparison among more than two groups. One-tailed *t*-test (parametric, unpaired with Welch’s correction) was performed for comparison between two groups. *P*-value < 0.05 was considered significant and depicted as * while *p*-value < 0.01 was depicted as ^**^. All the experiments were conducted at-least 3 times. Error bars indicate mean ± SEM.

## Results

### miR-128 expression is decreased in the exosomes derived from PD patient plasma

**FIGURE 1:**
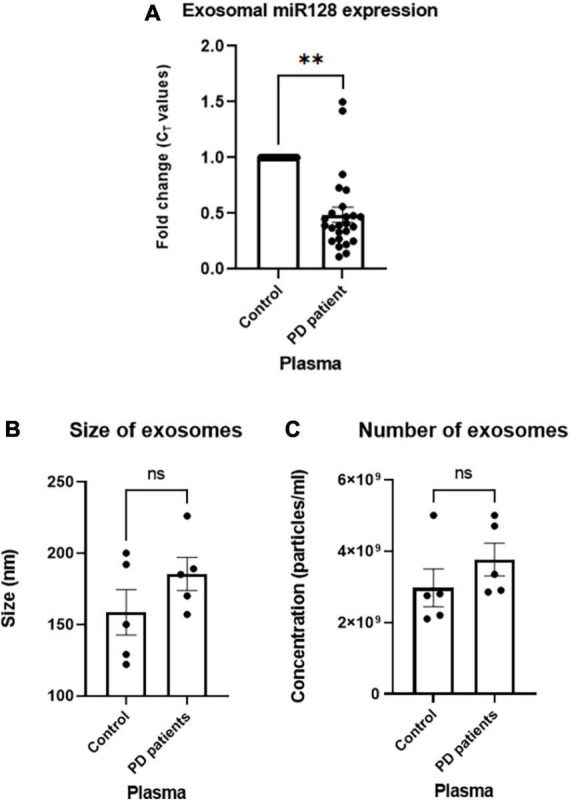
miR-128 expression decreases in the human Parkinson’s disease (PD) patient plasma-derived exosomes: **(A)** miR-128 expression in the exosomes derived from PD patient (age > 40) plasma samples (*n* = 25) as compared to age-matched controls (*n* = 20). U6 expression was used as endogenous control for normalization. **(B)** Nano-particle tracking analysis (NTA) analysis of the size of exosomes isolated from control (*n* = 5) and PD patient plasma (*n* = 5). **(C)** NTA analysis of the number of isolated exosomes from control (*n* = 5) and PD patient plasma (*n* = 5). Asterisks (*) indicate significant difference and “ns” indicates non-significant difference with respect to control, unless otherwise indicated in the figures. Data shown as mean ± SEM with ^**^p < 0.01.

First, we determined the prevalence of miR-128 in circulating sEV using EVAtlas (Liu et al., 2022) which is a comprehensive database for non-coding RNA expression in sEV obtained from human sRNA-Seq datasets. It showed a higher abundance of miR-128 in the total sEV derived from the plasma as compared to other body fluids (Supplementary Figure 1). Next, to validate this database generated result, we checked the expression of miR-128 in the exosomes derived from PD patient plasma of a local cohort. We collected blood samples from PD patients (age 40 and above) and respective age-matched controls (with no history of neurological disorder) and separated plasma by centrifugation. Next, intact exosomes were collected using miRCURY exosome isolation kit from serum/plasma as per manufacturer’s instructions (Helwa et al., 2017; López-Pérez et al., 2021) and the intra-exosomal total RNA was isolated. qRT-PCR based detection of the expression of miR-128 revealed a significant decrease in the expression of exosomal miR-128 obtained from PD patient subsets as compared to controls, as represented in Figure 1A. We further subjected the intact isolated exosomes to nano-particle tracking analysis (NTA) to confirm the size of the exosomes, as indicated in Figure 1B. Interestingly, there was no significant difference in the number of exosomes isolated from PD patient plasma in comparison to control (Figure 1C). This indicated that the difference in the level of exosomal miR-128 between the PD patient and control samples is due to a decrease in the expression of miR-128 and not due to any change in the quantity of exosomes released. Thus, our initial results showed that there is a significant reduction in the levels of the brain-enriched miR-128, as identified in the exosomes circulating in the blood of PD patients.

### miR-128 prevents neuronal death and inhibits mitochondrial superoxide production in cellular models of PD

miR-128 being a neuron-enriched miRNA, we next investigated if there is any intra-neuronal mechanistic significance of this reduced levels of miR-128 in context to PD. Since rodent dopaminergic neuron cultures have low purity, we used standard cellular models of PD, using human SH-SY5Y and rat PC12 cell lines which have reported to show dopaminergic neuronal properties upon priming with ATRA and NGF-β respectively (Falkenburger et al., 2016). We treated the cells with the neurotoxin 6-OHDA, a dopamine analog, that is up-taken by the dopamine transporters and is known to selectively damage the dopaminergic neurons (catecholaminergic) in the mid-brain (Simola et al., 2007).

**FIGURE 2:**
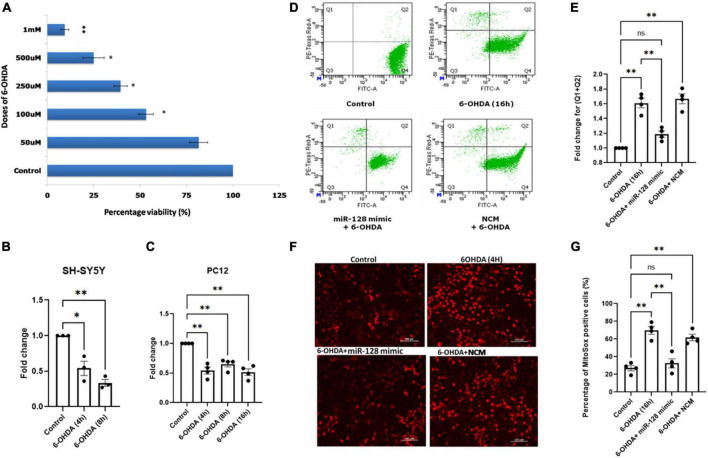
miR-128 prevents decrease in mitochondrial superoxide production and prevents death of neuronal cells *in vitro*: **(A)** Human SH-SY5Y cells were primed with 10 μM ATRA for 5 days and were then treated with varying doses (50 μM, 100 μM, 250 μM, 500 μM, and 1 mM) of 6-OHDA for 24 h respectively. MTT assay was done after 24 h and percentage viability of cells was calculated with respect to untreated (control) cells. **(B)** Primed SH-SY5Y cells were treated with 100 μM dose of 6-OHDA for 4 and 8 h respectively and miR-128 expression was checked by qRT-PCR with respect to control; (*n* = 3). **(C)** Rat PC12 cells were primed with 100 ng NGF-β for 5 days and were then treated with 100 μM dose of 6-OHDA for 4, 8, and 16 h respectively and miR-128 expression was checked by qRT-PCR with respect to control; (*n* = 4). U6 expression was used as endogenous control for normalization in both panels **(B,C)**. **(D)** Primed SH-SY5Y cells were either untreated (control) or treated with 100μM 6-OHDA for 16 h or pre-transfected with miR-128 mimic or negative control mimic (NCM) for 48 h before treatment with 6-OHDA (100μM) for 16 h. The cells were then incubated with calcein-AM and ethidium homodimer-1 dyes respectively and the percentage of live and dead cells were determined by flow cytometry. Q4 indicates live cells while (Q2 + Q1) represent total dying or dead cells respectively. **(E)** Graphical representation of the fold change of dying and dead cells (Q1 + Q2) as determined in panel **(D)**; (*n* = 4). **(F)** Primed SH-SY5Y cells were either untreated (control) or treated with 100 μM 6-OHDA for 4 h or pre-transfected with miR-128 mimic or NCM for 48 h before treatment with 6-OHDA (100 μM) for 4 h. The cells were incubated with MitoSOX dye and visualized under fluorescence microscope (20X). **(G)** Percentage of MitoSOX positive cells as calculated from panel **(F)**; (*n* = 4). Asterisks (*) indicate significant difference and “ns” indicates non-significant difference with respect to control, unless otherwise indicated in the figures. All experiments have been done in triplicate. Data shown as mean ± SEM with *p < 0.05 and ^**^p < 0.01.

First, we treated primed SH-SY5Y cells with varying doses of 6-OHDA and identified the D_50_ dose of 100 μM by MTT assay (Figure 2A). Next, we treated the cells with 100 μM dose of 6-OHDA for varying time points and checked the expression of miR-128 by qRT-PCR. Interestingly, there was a significant decrease in the expression of miR-128 by 4 and 8 h of 6-OHDA treatment (Figure 2B). To confirm that this result is not a cell-line specific, we repeated the experiment in primed PC12 cells with 100 μM dose of 6-OHDA (Sanphui et al., 2020) and found a similar decrease in miR-128 expression with increasing time-points of treatment (Figure 2C). This proved that 6-OHDA treatment results in a decrease in miR-128 expression in the neuronal models of PD. Depending on these results, for our subsequent experiments, we decided to choose 100 μM of 6-OHDA in the treatment window of 4–16 h on SH-SY5Y or PC12 cell lines. In all cases, we used four experimental conditions: untreated cells (control), cells treated with 6-OHDA for indicated time-points, cells pre-transfected with miR-128 mimic (over-expression constructs) or negative control mimic (NCM) for 48 h before treatment with 6-OHDA.

Firstly, we investigated whether miR-128 has an overall protective or detrimental role on neurons. We performed a flow cytometry-based live/dead cell assay. SH-SY5Y cells were treated with 6-OHDA for 16 h. The cells were then incubated with the green fluorescent dye calcein-AM (staining live cells) and the red fluorescent dye ethidium homodimer-1 (staining dead cells) and the percentage of live/dead cells was determined by flow cytometry. There was a significant decrease in live cells (Q4) and increase in total dying or dead cells (Q2 + Q1) with 6-OHDA treatment which was significantly reversed in cells pre-transfected with miR-128 mimic (Figure 2D). The change in the total percentage of dying and dead cells (Q2 + Q1) is expressed in Figure 2E. These results indicate that miR-128 can protect against neuronal death.

Next, we decided to check the effect of miR-128 on mitochondrial superoxide production. Mitochondria gets affected at a relatively early stage preceding neuronal death, so we treated SH-SY5Y cells with 6-OHDA for 4 h for this experiment. We performed MitoSOX assay for detection of mitochondrial superoxide production in live cells under fluorescent microscope. As compared to the control, there was an increase in superoxide production indicated by increase in red fluorescence in the 6-OHDA treated cells which was significantly reduced in the cells pre-transfected with miR-128 mimic. No such reduction in fluorescence was observed in the cells transfected with the NCM (Figure 2F). An arbitrary unit (A.U.) of red fluorescence was assigned as threshold such that cells with equal or higher fluorescence intensity were considered MitoSOX positive and percentage of MitoSOX positive cells were calculated (Figure 2G). Thus, our results indicated that miR-128 has an overall neuroprotective function whereby it not only prevented mitochondrial superoxide production but also protected against 6-OHDA mediated cell death.

### miR-128 prevents both intrinsic and extrinsic pathways of apoptosis downstream of FoxO3a activation

**FIGURE 3:**
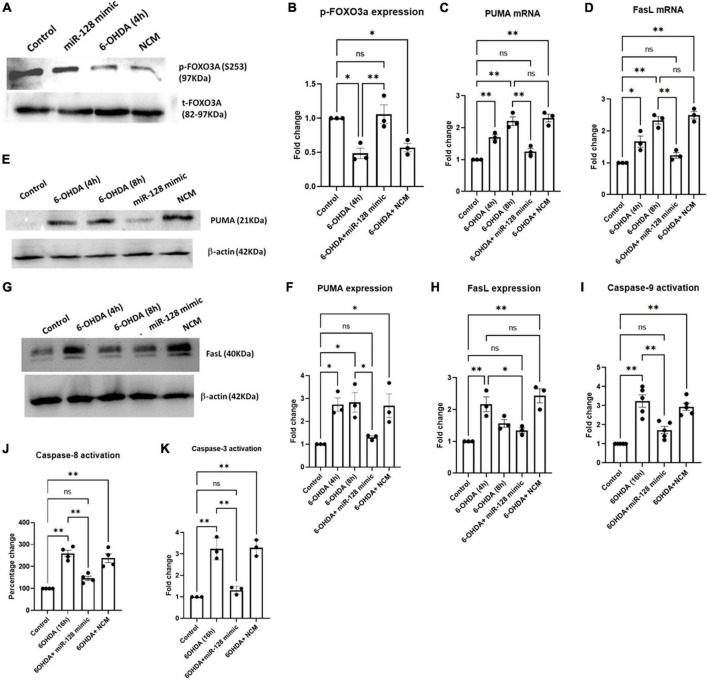
miR-128 regulates activation of FOXO3a and prevents neuronal death *via* both intrinsic and extrinsic apoptotic pathways *in vitro*: Primed SH-SY5Y cells were either untreated (control) or treated with 100 μM 6-OHDA for indicated time-points or pre-transfected with miR-128 mimic or negative control mimic (NCM) for 48 h before treatment with 6-OHDA (100 μM) for the respective time-points, in all the experiments. **(A)** Total protein lysates were subjected to immunoblotting against p-FOXO3a (Ser253) and total FOXO3a. **(B)** Graphical representation of the immunoblots as represented in panel **(A)**; (*n* = 3). Normalization was done against total FOXO3a. **(C,D)** Total RNA was isolated from the cells and mRNA levels of PUMA (*n* = 3) and FasL (*n* = 3) was measured by qRT-PCR. GAPDH expression was used as endogenous control for normalization. **(E,G)** Total protein lysates were subjected to immunoblotting against PUMA and FasL respectively. β-actin protein expression was used as endogenous control for normalization in both cases. **(F,H)** Graphical representations of protein expressions of PUMA (*n* = 3) and FasL (*n* = 3) as represented in panels **(E,G)** respectively. **(I)** Cell lysate was incubated with the chromophore labeled substrate (LEHD–*p*NA) and O.D. was measured at 405 nm; (*n* = 4). **(J)** Cells were incubated with FLICA reagent and fluorescence end point reading was detected at E_x_ = 490 nm and E_m_ = 520 nm; (*n* = 4). **(K)** Cell lysate was incubated with the chromophore labeled substrate (LEHD–*p*NA) and O.D. was measured at 405 nm; (*n* = 3). Asterisks indicate significant difference and “ns” indicates non-significant difference with respect to control, if not otherwise indicated in the figure. All experiments were done at least in triplicate. Data shown as mean ± SEM with *p < 0.05 and ^**^p < 0.01.

Apoptosis being one of the major pathways of neuronal death in PD, we explored the involvement of miR-128 in the apoptotic pathways. We have previously shown in AD models that activation of the transcription factor FoxO3a is a major inducer of apoptotic death in neurons (Akhter et al., 2014). We have also reported activation of FoxO3a in PC12 cells under 6-OHDA treatment (Sanphui et al., 2020). Thus, we investigated whether miR-128 exhibited its neuroprotective effect *via* regulating activation of FoxO3a. FoxO3a activation involves its translocation from the cytosol to the nucleus which is triggered by specific post-translational modifications (Nho, 2014). In the deactivated state, FoxO3a is phosphorylated at Ser 253 position and is retained in the cytoplasm. Dephosphorylation of FoxO3a at Ser 253 triggers activation signals whereby FoxO3a is translocated to the nucleus. Thus, a reduction of its phosphorylated form at Ser 253 indicates activation of FoxO3a. We treated SH-SY5Y cells with 6-OHDA for 4 h and subjected the protein samples to immunoblotting against p-FoxO3a (Ser 253) and total FoxO3a. In the 6-OHDA treated samples, there was a marked reduction in the p-FoxO3a (Ser 253) levels, indicating FoxO3a activation which was significantly reversed in miR-128 mimic transfected cells. The total FoxO3a levels remained relatively unchanged throughout (Figures 3A, B). This indicated that miR-128 can prevent activation of the transcription factor FoxO3a.

Two major signaling cascades- the extrinsic and intrinsic pathways of apoptosis, involve up regulation of pro-apoptotic proteins FasL and PUMA respectively, at the initial stages of apoptotic induction (Elmore, 2007). FoxO3a has been reported to induce expression of both PUMA and FasL in response to various apoptotic stimuli (Nho, 2014). Since miR-128 was found to regulate FoxO3a activation, we decided to check whether miR-128 can regulate the induction of apoptotic pathways downstream of FoxO3a. We treated SH-SY5Y cells with 6-OHDA for 4 and 8 h respectively and checked the expression of PUMA and FasL mRNAs by qRT-PCR. A marked increase of both PUMA and FasL transcript levels was observed by 4 h of 6-OHDA treatment which was significantly reduced by pre-transfection with miR-128 mimic (Figures 3C, D). These results were further validated when immunoblotting showed similar reduction in the expression of PUMA (Figures 3E, F) and FasL (Figures 3G, H) upon miR-128 over-expression.

To further validate the involvement of miR-128 in apoptotic pathways, we checked the effect of miR-128 on the irreversible activation of downstream caspases. Intrinsic and extrinsic pathways of apoptosis involves activation of caspases- 9 and -8 respectively, both of which lead to the activation of caspase-3, thus eventually resulting in apoptosis (Fan et al., 2005). By using colorimetic assay, we observed an increase in the caspase-9 activity with 6-OHDA treatment which was subsequently reduced by over-expression of miR-128 (Figure 3I). Next, we performed a fluorescence-based assay for caspase-8 activity and detected an increase in the caspase activity upon 6-OHDA treatment which was significantly reduced by pre-treatment with miR-128 mimic (Figure 3J). Lastly, we performed a colorimetric caspase-3 activity assay which indicated that miR-128 over-expression could attenuate 6-OHDA mediated activation of caspase-3 as well (Figure 3K). Thus, our results comprehensively indicate that miR-128 prevents both the apoptotic pathways- PUMA-mediated intrinsic and FasL-mediated extrinsic, while also inhibiting the upstream activation of FoxO3a.

### miR-128 prevents neurite shortening and maintains pre- and post-synaptic protein expressions

**FIGURE 4:**
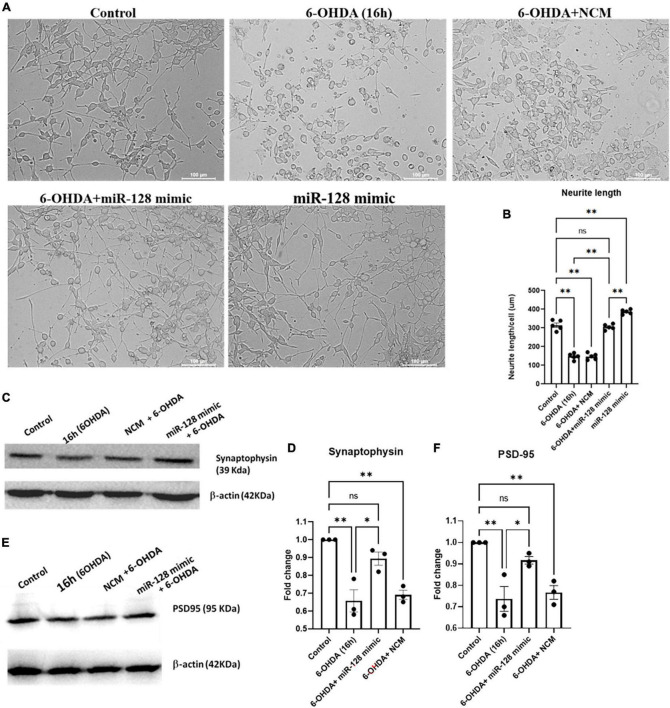
miR-128 improves neurite length and maintains pre- and post-synaptic protein expression *in vitro*: **(A)** Primed PC12 cells were either untreated (control) or treated with 6-OHDA (100 μM) for 16 h or pre-transfected with miR-128 mimic or negative control mimic (NCM) for 48 h before treatment with 6-OHDA (100 μM) for 16 h. The cells were observed under bright-field microscope (20X). **(B)** Neurite length (μM) per 50 cells per field was calculated using Neuron J plugin of Fiji software; (*n* = 5). **(C,E)** Primed SH-SY5Y cells were either untreated (control) or treated with 100 μM 6-OHDA for 8 h or pre-transfected with miR-128 mimic or NCM for 48 h before treatment with 6-OHDA (100 μM) for 8 h. Total protein lysates were subjected to immunoblotting against synaptophysin panel **(C)** and PSD-95 panel **(E)**. β-actin protein expression was used as endogenous control for normalization in both cases. **(D,F)** Graphical representations of protein expressions of synaptophysin panel **(C)** and PSD-95 panel **(E)** as normalized with respect to β-actin expression; (*n* = 3 for each). Asterisks (*) indicate significant difference and “ns” indicates non-significant difference with respect to control, unless otherwise indicated in the figures. All experiments were done in triplicate. Data shown as mean ± SEM with *p < 0.05 and ^**^p < 0.01.

miR-128 being enriched at the neurites and synaptic vesicles (Franzoni et al., 2015; Kumar and Reddy, 2020), we decided to check the effect of miR-128 on neurite formation and synaptic integrity respectively. We treated primed PC12 cells with 6-OHDA for 16 h and observed their morphology under bright-field microscope. As compared to the control, a marked reduction in neurite lengths under 6-OHDA treatment was observed. However, this shortening of neurite lengths was significantly reversed by miR-128 over-expression whereby the overall morphology of the primed neuronal cells was maintained (Figure 4A). Neurite length/cell (μm) was measured by Neuron J plugin of Fiji software (Figure 4B).

Next, we investigated whether miR-128 has any effect on synaptic protein expressions. We treated SH-SY5Y cells with 6-OHDA for 16 h and checked the expression of the synaptic proteins synaptophysin and PSD-95 by immunoblotting. By 16 h of 6-OHDA treatment we observed a significant decrease in the levels of both synaptophysin (Figures 4C, D) and PSD-95 (Figures 4E, F). Interestingly, this decrease in synaptic protein expression was attenuated by over-expression of miR-128. Thus, our results showcase that miR-128 can not only maintain the overall neuronal morphology by preventing neurite shortening, but also keep the synaptic integrity intact by maintaining the expression of pre- and post-synaptic proteins like synaptophysin and PSD-95 respectively.

## Discussion

In the present study, we investigated the role of a particular neuron-enriched miRNA, miR-128, in PD pathogenesis. Our study identifies miR-128 to be significantly decreased in the exosomes derived from the PD patient plasma as compared to age-matched control subsets. This characteristic decrease in the circulating exosomal miR-128 expression was further corroborated with a similar reduction in miR-128 expression in a standard cellular model of PD. We also depicted detailed mechanistic implications of this altered miR-128 expression in the pathogenesis of PD. Interestingly, as per a study by Hanan et al. (2020), an increase in the brain-enriched circular RNA circSLC8A1 was found in the SN region of PD patients. CircSLC8A1 was shown to carry 7 binding sites for miR-128. As a result, the levels of mRNA targets of miR-128 were also increased in the PD patients, thereby indicating that circSLC8A1 may regulate/sponge miR-128 function and activity. Additionally, CircSLC8A1 levels were also found to increase in cell culture models upon Paraquat treatment. All these results indirectly support our results where we found a decrease in the miR-128 levels in cell culture models as well as in the exosomes derived from the PD patient plasma while there was a concomitant increase in the downstream targets of miR-128 like FasL and PUMA in the 6-OHDA treated culture models.

So far, only a handful of reports have come out suggesting the role of miR-128 in PD. For instance, miR-128 was reported to regulate AXIN1 and protect dopaminergic neurons from apoptosis in models of PD (Zhou et al., 2018). Another report showed miR-128 expression to be reduced in hippocampal tissue of MPTP mouse models (Zhang et al., 2020). They reported that HIF-1α/miRNA-128-3p axis is neuroprotective *via* the Axin1-mediated Wnt/β-catenin signaling pathway in models of PD. miR-128 is also extensively reported as a regulator of nonsense-mediated decay (Bruno et al., 2011; Karam and Wilkinson, 2012; Karam et al., 2013). It is worth mentioning here that although miR-128 has been predominantly reported to be neuroprotective in nature (Mao et al., 2017; Fang et al., 2019; Shi et al., 2021), it is also shown to promote neuronal degeneration under certain conditions (Adlakha and Saini, 2013; Geng et al., 2018). However, a detailed mechanistic study on the involvement of miR-128 in the apoptotic pathways of neuronal death associated with PD was still largely unexplored.

This study showcases a comprehensive mechanistic function of miR-128 in regulating the molecular pathways leading to neurodegeneration in PD. In cellular models of PD, miR-128 was shown to have neuroprotective functions whereby it regulated the activation of the transcription factor FoxO3a and prevented 6-OHDA induced neuronal apoptosis further downstream. Within the nucleus, FoxO3a is instrumental in activating apoptotic pathways, under various conditions of cellular stress (Nho, 2014). Apoptosis, a major cell death mechanism, maybe induced through two major pathways- the intrinsic and the extrinsic pathways (Elmore, 2007). One of the pro-apoptotic proteins found to be up-regulated in the early stages of intrinsic and extrinsic apoptotic pathways is PUMA and FasL respectively, both being direct targets of activated FoxO3a. Interestingly, miR-128 was found to not only regulate FoxO3a activation, but also regulate the expressions of both PUMA and FasL, downstream of FoXO3a. Finally, miR-128 could prevent the activation of caspases-8, -9 and -3, eventually shutting down both the intrinsic and extrinsic pathways of apoptosis. In addition to that, mitochondrial dysfunction is known to be associated with both the sporadic and familial forms of PD and various interventions targeting mitochondrial impairment and superoxide production have been reported in the past (Luo et al., 2015; Gao et al., 2022). Interestingly, our study revealed that miR-128 over-expression could be an important strategy in controlling mitochondrial superoxide production during PD pathogenesis.

Being a neurodegenerative disorder, PD is also associated with synaptic dysregulation preceding neuronal death. Additionally, miR-128 has been detected in synaptic vesicles and has been found to be enriched at the neurites (Franzoni et al., 2015; Kumar and Reddy, 2020). Our results depicted that miR-128 over-expression can prevent down-regulation of pre-synaptic terminal protein Synaptophysin and post-synaptic density protein PSD-95 although we were unable to find a direct molecular target through which miR-128 could regulate the expression of these synaptic proteins, in our models of study. Interestingly enough, miR-128 could also inhibit 6-OHDA induced neurite shortening while over-expression of miR-128 showed improved neurite formation. Our observation was consistent with that of Evangelisti et al. (2009) who showed that ATRA mediated neurite formation in SH-SY5Y cells is associated with miR-128 upregulation. Sun et al. (2013) too reported that in rat primary cortical neurons, inhibitors of miR-128 could block the neurite growth promoting effects of HspB1.

In the past, there have been reports on the presence of a wide range of miRNAs in the circulating exosomes of PD patient cohorts (Gui et al., 2015; Cao et al., 2017; Yao et al., 2018; Manna et al., 2021). But to our knowledge, a detailed and targeted study on dysregulated brain-enriched miRNAs, like miR-128, obtained from the exosomes of PD patients plasma has not been done till date. Although it needs validation in large and varied patient pool to test its candidacy as a potential biomarker for PD, our study cohesively proves the importance of miR-128 in pathogenesis of PD at both the extracellular and intra-neuronal states. Overall, through our results obtained from human patient samples and cellular models, we suggest that the altered expression of circulating exosomal miR-128 could be an important player in PD pathogenesis and a target for detection of PD.

## Conclusion

In recent times, there have been some important reports that depict the significance of brain-enriched and neuron-enriched miRNAs as biomarkers of brain-associated pathologies like PD and traumatic brain injury (Sheinerman et al., 2017; O’Connell et al., 2020; Ravanidis et al., 2020). In fact, brain-enriched miRNAs have shown to possess a very high accuracy of upto 96% in detecting Mild Cognitive Impairment, as reported by DiamiR, a molecular diagnostic company for detection of neuropathies^1^ (Sheinerman et al., 2012). Additionally, the identification of brain-enriched miRNAs and their subsequent correlation with exosome subtypes and the association of the exosome subtypes to a particular function and/or group of functions within the cell, along with the potential for new technologies to engineer target-specific exosomal particles, could pave the way for effective application of exosomes in clinical use. In the present study, we have showed how neuron-enriched miR-128 is an important regulator of neurodegeneration and is significantly implicated in the neuropathology of PD. Through focusing on miR-128, our work highlights the importance of dysregulated brain-enriched miRNAs, easily detectable in circulating exosomes of patient blood, in bridging the gap in the detection, diagnosis and treatment of incurable brain disorders like PD.

## Data availability statement

The original contributions presented in this study are included in the article/Supplementary material, further inquiries can be directed to the corresponding author.

## Ethics statement

The studies involving human participants were reviewed and approved by Human Ethics Committee of CSIR-Indian Institute of Chemical Biology. The patients/participants provided their written informed consent to participate in this study.

## Author contributions

PB conceived and designed the study and performed all biochemical, molecular, and cellular experiments. SB supervised the project. AB performed the clinical assessment of patients and collection of blood. PB and SB analyzed the data and contributed to writing the manuscript. All authors read and approved the final submitted manuscript.

## Footnotes

1
https://www.diamirbio.com/
