# Supplementary material for: Brain-enriched miR-128: Reduced in exosomes from Parkinson’s patient plasma, improves synaptic integrity, and prevents 6-OHDA mediated neuronal apoptosis
Source: Front Cell Neurosci. 2023 Jan 12;16:1037903. doi: 10.3389/fncel.2022.1037903 (PMC9879011; doi:10.3389/fncel.2022.1037903)
Supplement: Supplementary file 1 [file Data_Sheet_1.docx]

**Supplementary figures**


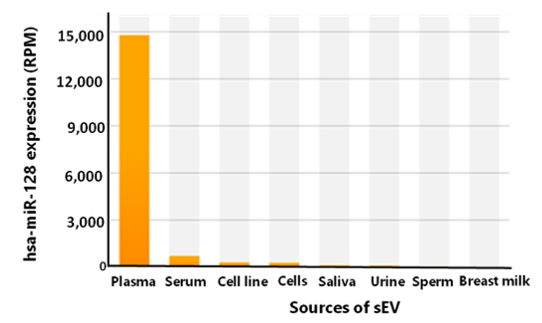


**Fig. S1: Human small RNA-Seq based determination of miR-128 abundance from various sources:** miR-128 expression in the sEV derived from different sources in the EVAtlas database. miRNA expression is plotted in RPM values. All results were represented in reads per million (RPM) which indicates high abundance (> 100 RPM), medium abundance (10–100 RPM) or low abundance (1–10 RPM).

**Supplementary tables**

**Table ST1: List of miRNA reagents**

| **miRNA assay** | **Catalog number (Ambion)** |
| --- | --- |
| hsa-miR-128a | 4427975  Assay ID: 002216 |
| U6 | 4427975  Assay ID: 001973 |
| miR-128 mimic | [MC11746](https://www.thermofisher.com/order/genome-database/details/microrna/MC11746?CID=&ICID=&subtype=microrna_mimics_inhibitors) |
| Negative control mimic | 4464058 |

**Table ST2: List of primers**

| **mRNA primers** | **Sequence** |
| --- | --- |
| PUMA forward primer | 5’ACGACCTCAACGCACAGTACGA3’ |
| PUMA reverse primer | 5’CCTAATTGGGCTCCATCTCGGG3’ |
| FasL forward primer | 5’GGTTCTGGTTGCCTTGGTAGGA3’ |
| FasL reverse primer | 5’CTGTGTGCATCTGGCTGGTAGA3’ |
| GAPDH forward primer | 5’TCAACAGCAACTCCCACTCTT3’ |
| GAPDH reverse primer | 5’ACCCTGTTGCTGTAGCCGTAT3’ |

**Table ST3: List of primary antibodies**

| **Primary antibodies** | **Catalog number** |
| --- | --- |
| p-FoxO3a (Ser253) | Cell Signaling Technology (9466) |
| FoxO3a | Cell Signaling Technology (2497) |
| PUMA | Novus Biologicals (NBP1-76639) |
| FasL | Santa Cruz Biotechnology(sc-6237) |
| PSD-95 | Santa Cruz Biotechnology (sc-32290) |
| Synaptophysin | Novus Biologicals (NBP2-25170) |
| β-Actin | Sigma-Aldrich (A3854) |

**Table ST4: Details of human PD patients studied**

| Number of age-matched controls | 20 |
| --- | --- |
| Number of PD patients | 25 |
| Number of female PD patients | 07 |
| Number of male PD patients | 18 |
| Average age group of PD patients | 40-70 |
| Number of sporadic PD | 17 |
| Number of familial PD | 08 |

**Supplementary Material & Methods**

**Human small RNA-Seq data**

For human small RNA-Seq (sRNA-Seq) data, resources from EVAtlas (<http://bioinfo.life.hust.edu.cn/EVAtlas>) were used.

**MTT assay**

MTT powder was dissolved in culture medium and added to cells *in vitro* at a final concentration of 0.5mg/ml. Cells were then incubated for 3-4 hours in the humidified cell culture incubator (37^0^C, 5% CO_2_) until purple formazan crystals were visible. The culture medium was carefully discarded and replaced with DMSO to dissolve the formazan crystals in an orbital shaker for 15-20 minutes. Optical density (O.D.) was measured at 570nm.

**Caspase-8 fluorimetric assay**

Caspase-8 activity was determined using CaspaTag Caspase-8 In Situ Assay Kit (Merck Millipore) following manufacturer’s protocol. Briefly, fluorochrome inhibitors of caspases (FLICA reagent) was used which produces a green signal indicating the amount of active caspase-8 inside the cells. End-point reading was obtained in a multi-plate reader (Thermo Scientific) using excitation at 490nm and emission at 520nm.

**Caspase-9 colorimetric assay**

Caspase-9 activity was determined using Caspase-9 Colorimetric Activity Assay Kit (Merck Millipore) following manufacturer’s protocol. Spectrophotometric detection of the chromophore product p-nitroaniline was done at 405nm in a multi-plate reader (Thermo Scientific). Fold change in the caspase-9 activity in the cells was calculated from the obtained O.D.

**Caspase-3 colorimetric assay**

Caspase-3 activity was determined using Caspase-3 colorimetric Assay Kit (Sigma-Aldrich) following manufacturer’s protocol. Briefly, the hydrolysis of the peptide substrate by active caspase-3 resulted in the release of the chromophore product p-nitroaniline which was detected at 405nm using a multi-plate reader (Thermo Scientific). From the obtained readings, fold change in the caspase-3 activity was calculated.
